# Down‐regulation of the mitochondrial i‐AAA protease Yme1L induces muscle atrophy via FoxO3a and myostatin activation

**DOI:** 10.1111/jcmm.14799

**Published:** 2019-11-14

**Authors:** Yoo Jeong Lee, Gyu Hee Kim, Sang Ick Park, Joo Hyun Lim

**Affiliations:** ^1^ Division of Endocrine and Metabolic Disease Center for Biomedical Sciences Korea National Institute of Health Cheongju Korea

**Keywords:** mitochondria quality control, muscle atrophy, Yme1L

## Abstract

Muscle atrophy is closely associated with many diseases, including diabetes and cardiac failure. Growing evidence has shown that mitochondrial dysfunction is related to muscle atrophy; however, the underlying mechanisms are still unclear. To elucidate how mitochondrial dysfunction causes muscle atrophy, we used hindlimb‐immobilized mice. Mitochondrial function is optimized by balancing mitochondrial dynamics, and we observed that this balance shifted towards mitochondrial fission and that MuRF1 and atrogin‐1 expression levels were elevated in these mice. We also found that the expression of yeast mitochondrial escape 1‐like ATPase (Yme1L), a mitochondrial AAA protease was significantly reduced both in hindlimb‐immobilized mice and carbonyl cyanide m‐chlorophenylhydrazone (CCCP)‐treated C2C12 myotubes. When Yme1L was depleted in myotubes, the short form of optic atrophy 1 (Opa1) accumulated, leading to mitochondrial fragmentation. Moreover, a loss of Yme1L, but not of LonP1, activated AMPK and FoxO3a and concomitantly increased MuRF1 in C2C12 myotubes. Intriguingly, the expression of myostatin, a myokine responsible for muscle protein degradation, was significantly increased by the transient knock‐down of Yme1L. Taken together, our results suggest that a deficiency in Yme1L and the consequential imbalance in mitochondrial dynamics result in the activation of FoxO3a and myostatin, which contribute to the pathological state of muscle atrophy.

## 1 INTRODUCTION

Muscle atrophy is now considered to be closely associated with many diseases, including cardiac failure and diabetes 1; however, the underlying mechanisms still unclear. Muscle atrophy primarily accompanied by protein breakdown in muscles; in particular, the ubiquitin‐proteasome system (UPS) is most prominent mechanism in this process. In particular, increased expression of muscle‐specific E3 ubiquitin ligases, such as muscle RING finger 1 (MuRF1) and muscle atrophy F‐box (MAFbx)/atrogin‐1, is a typical feature of muscle atrophy.^2^ Recently, muscle was proposed to be an endo/paracrine organ that secretes several proteins called myokines.^3^ Myostatin is one of the major myokines, which belongs to the transforming growth factor β (TGF β) superfamily and is known to substantially increase muscle protein degradation via the MuRF1 or atrogin‐1 mediated ubiquitin‐proteasome system.^4^ Additionally, the activation of forkhead box O3 (FoxO3) is considered a key pathway that mediates muscle protein degradation.^5^ When FoxO3a is phosphorylated by Akt, its activity is inhibited. In contrast, the phosphorylation of FoxO3a via AMPK or JNK promotes the activity of FoxO3a. Inhibition of the IGF‐1 (insulin‐like growth factor‐1)/Akt/mTOR pathway and elevation of AMPK together facilitate FoxO3a activity, which concomitantly leads to muscle atrophy.

Growing evidence has shown that mitochondrial dysfunction is one of the important factors in muscle atrophy.^6^ Mitochondria are dynamic organelles, and their functions are maintained by mitochondrial quality control systems which comprise mitochondrial biogenesis, fusion, fission, proteostasis and mitophagy. A balance between these procedures is important for the maintenance of mitochondrial homeostasis, and conditions that shift the balance towards mitochondrial fission lead to mitochondrial fragmentation.^7^ Three different dynamin‐related guanosine triphosphatases (GTPases) are involved in mitochondrial fusion: mitofusin 1 (Mfn1) and mitofusin 2 (Mfn2) participate in the fusion of the outer mitochondrial membrane (OMM) and optic atrophy 1 (Opa1) participates in the fusion of the inner mitochondrial membrane (IMM). Opa1 has various isoforms, eight in humans and four in mice, and the long form (L‐Opa1) and short form (S‐Opa1) of Opa1 are generated by alternative splicing or proteolytic cleavage. Under normal conditions, these forms exist at almost equimolar concentrations, and the loss of L‐Opa1 leads to mitochondrial fragmentation.^8^ Mitochondrial fission requires the recruitment of dynamin‐related protein 1 (Drp1), another type of dynamin‐related GTPase, to the OMM and the formation of multiple ring complexes. Mitochondrial fission protein 1 (Fis1), mitochondrial fission factor (Mff) and mitochondrial dynamics proteins of 49 kD (MID49) and 51 kD (MID51) act as receptors for Drp1 at the OMM.

To ensure proper function, mitochondria contain numerous chaperones and proteases in each mitochondrial subcompartment.^9^ Similar to endoplasmic reticulum‐associated protein degradation (ERAD), mitochondria generate an unfolded protein response (mtUPR) in response to stress conditions to restore normal functions by mitochondrial proteostasis and chaperone. Depending on subcompartment localization, mitochondrial proteases are divided into four different groups: mitochondrial matrix proteases, inner membrane (IM) proteases, intermembrane space (IMS) proteases and outer membrane (OM) proteases. Mitochondrial processing peptidase (MPP), LonP and ClpP, are representative matrix protease. Several IMS proteases, such as presenilin‐associated rhomboid‐like (PARL) and HTRA2, together with USP 30 located in the OM, participate in mitophagy, a specialized autophagy that eliminates irreversibly damaged mitochondria.^10^ Mitochondrial ATPases associated with diverse cellular activities (AAA) proteases located in the IM are crucial for mitochondrial homeostasis and are subdivided into intermembrane space AAA (i‐AAA) proteases and matrix AAA (m‐AAA) proteases depending on the location of their active sites. In humans, m‐AAA proteases are homo‐ or hetero‐oligomeric proteins composed of ATPase family gene 3‐like protein 2 (AFG3L2) and paraplegin (Spg7), whereas i‐AAA proteases consist of homo‐oligomeric Yme1L proteins.^11^ Together with AAAs, the ATP‐independent peptidase Oma1, also located in the IM, participates in mitochondrial dynamics through the processing of Opa1.^12^

In this study, we demonstrated that muscle atrophy was closely related to an imbalance in the mitochondrial quality control system. In particular, a deficiency in Yme1L accelerated this imbalance towards mitochondrial fission and mitophagy, subsequently activating FoxO3a and myostatin, which are essential for the degradation of muscle proteins. Apparently, Yme1L plays an important role in the maintaining healthy mitochondria in the muscle and loss of Yme1 induces the pathological state of muscle atrophy.

## 2 MATERIALS AND METHODS

### 2.1 Animal studies

Male C57BL/6 mice (7 weeks old) were purchased from Koatech (Pyeongtaek, Korea) and were housed at a constant room temperature (23°C) with a 12 hours light/dark cycles and free access to food and water. After a week of adaptation, the animals were randomly assigned to two groups: Control group (n = 8) and hindlimb immobilization (disuse) group (n = 8). For the induction of muscle atrophy, the hindlimbs of the mice were immobilized by stapling the foot to the limb using a surgical stapler (Visistat Skin Stapler 35W, WECK, Teleflex), as previously described.^13^ After 5 days of immobilization, the animals were anaesthetized, and then, the gastrocnemius muscles (GA) were dissected and immediately frozen in liquid nitrogen for subsequent analyses. All animal procedures were performed according to the Korean National Institutes of Health Animal Care and Use Committee (Permit Number: KCDC‐061‐18‐2A), and the animals were cared for in accordance with the Korea National Institute of Health guidelines.

### 2.2 Grip strength and rotarod test

Five days after immobilization, forelimb skeletal muscle strength was measured using a digital grip strength metre (Bioseb). The mean of three consecutive trials was considered an index of grip strength. For the rotarod test (Med‐associated), the mice were placed on a rotating rod that accelerated from 4 to 40 rpm over 5 minutes.^14^ The mean time to fall from the rod (s) in three different trials was analysed individually.

### 2.3 Measurement of myosin heavy chain distribution

Serial paraffin‐sections of GA muscle were deparaffinized and permeabilized with 1% Triton X‐100 for 5 minutes before the samples were blocked with 10% normal goat serum. Then sections were stained with the following antibodies: SC‐71 for the Type 2a MyHC isoform, BF‐F3 for the 2b MyHC isoform and BA‐F8 for the Type 1 MyHC (Hybridoma Bank). Primary antibodies were detected by anti‐mouse IgG Alexa Fluor‐647 conjugate, anti‐mouse IgM Alexa Fluor‐568 conjugate, or anti‐mouse IgG2b Alexa Fluor‐488 (eBioscience; Thermo Fisher Scientific, Inc) for visualization, respectively. Fluorescence was detected using an FV3000 laser scanning confocal microscope (Olympus Corporation).

### 2.4 Cell culture and Yme1L small interfering RNA transfection

Murine C2C12 myoblasts were purchased from the American Type Culture Collection (ATCC) and maintained in medium consisting of Dulbecco's modified Eagle's medium (DMEM; Gibco; Thermo Fisher Scientific, Inc) supplemented with 1% penicillin‐streptomycin (Gibco)/ 10% FBS (Gibco) in 5% CO_2_ at 37°C. For the induction of myogenic differentiation, the cells were differentiated into myotubes with DMEM supplemented with 2% horse serum for 5 days. To induce mitochondrial dysfunction, the C2C12 myotubes were treated with 2, 5, or 10 μmol/L of the protonophore carbonyl cyanide m‐chlorophenylhydrazone (CCCP) for 24 hours. In the case of antioxidant treatment, the myotubes were pretreated with 2 mmol/L N‐acetylcysteine (NAC; Sigma)) or 0.5 μmol/L Mitoquinone mesylate (MitoQ; MedKoo Biosciences). For the siRNA transfection, the cells were transfected with control or gene‐specific siRNA 3 days after differentiation using Lipofectamine RNAiMAX (Invitrogen; Thermo Fisher Scientific, Inc) according to the manufacturer's protocol. The following sequences were used: 5′‐GGAUGCCAAAGAGAUUCAAAUUGUU‐3′ (murine Yme1L1); 5′‐GUAGGACUCUCAAUCACAATT‐3′ (murine Oma1); and 5′‐ CCACACAAGGCAAGAUCCUCUGCUU‐3′ (murine LonP1). The day after transfection, the medium was replaced with fresh DMEM containing 2% horse serum, and the cells were further incubated for 48 hours.

### 2.5 Determination of mitochondrial membrane potential

The mitochondrial membrane potential of C2C12 myotubes was assessed with a JC‐1 mitochondrial membrane potential assay kit (Abcam, ab113850) according to manufacturer's instructions. The cells were incubated with 10 μmol/L JC‐1 dye for 20 minutes at 37°C and then washed twice with 1X dilution buffer. The samples were assessed on a fluorescent plate reader (Molecular Device) with a set excitation wavelength of 535 nm and an emission wavelength of 590 nm.

### 2.6 Western blot analysis

Following CCCP treatment or transfection with siRNA, whole cell lysates (30 µg) were prepared with 25 µL of SDS‐PAGE sample buffer and loaded on 4%‐12% SDS‐PAGE gels. Antibodies against phospho‐FoxO3a (S253) (#9466), FoxO3a (#2497), GAPDH (#2118), VDAC1 (#4661), Beclin 1 (#3495), p62 (#5114), LC3 I/II (#3868), phospho‐AMPK (#2535), AMPK (#2532), phospho‐p38 (#4511), phospho‐IR (#3024) and phospho‐AKT (S473) (#9271) were purchased from Cell Signaling Technology. Antibodies against Mfn1 (ab57602) and Mfn2 (ab56889) were obtained from Abcam. Antibodies against MuRF1 (sc‐398608), atrogin‐1 (sc‐166806), Fis1 (sc‐376447) and Oma1 (sc‐515788) were purchased from Santa Cruz Biotechnology, OXPHOS cocktail (45‐8099) and Yme1L (PA‐43806) were obtained from Thermo Fisher Scientific, and antibodies against Opa1 (612606) and Drp1 (611738) were purchased from BD Bioscience.

### 2.7 Quantitative PCR analysis

Total RNA was extracted from differentiated C2C12 myotubes or gastrocnemius muscle tissue using the RNeasy Mini kit (Qiagen) according to the manufacturer's instructions. cDNA was synthesized using SuperScript III reverse transcriptase (Invitrogen; Thermo Fisher Scientific, Inc). Quantitative PCR was performed using SYBR Green Master Mix (Thermo Fisher Scientific) in a total volume of 20 µL with the QuantStudio 6 Flex system (Thermo Fisher Scientific). The expression of the target genes was normalized to GAPDH expression, and relative quantification was evaluated with the 2^−ΔΔCt^ method. The primer pairs for the specific target genes were designed as listed in Table S1.

### 2.8 Immunofluorescence and reactive oxygen species measurement

Differentiated C2C12 cells were washed with PBS, fixed with 4% paraformaldehyde in PBS for 10 minutes and then permeabilized with 0.2% Triton X‐100 for 10 minutes at room temperature. The cells were then blocked with 2% BSA in PBS and incubated with an MF‐20 anti‐MHC Alex Fluor 488 antibody (eBioscience; Thermo Fisher Scientific, Inc) overnight. Images of microtubes were taken using a FV1000 laser scanning confocal microscope (Olympus, Japan).

To measure the formation of intracellular reactive oxygen species (ROS) in CCCP‐treated cells, C2C12 myotubes were incubated in serum‐free media containing 10 µmol/L 2′,7′‐dichlorofluorescin‐diacetate (DCF‐DA) cell permeant reagent (Invitrogen) for 20 minutes, and the cells were subsequently washed 3 times with PBS. DCF fluorescence images were obtained using a FV1000 laser scanning confocal microscope (Olympus, Japan).

### 2.9 Statistical analysis

All results are expressed as the means ± standard error of the mean (SEM). Statistical analysis was performed using Student's *t* tests with GraphPad Prism software (GraphPad). For multiple comparison, one‐way ANOVA (analysis of variance) followed up by Bonferroni's multiple comparison test were used to analyse statistical differences. Values of *P* < .05 were considered statistically significant.

## 3 RESULTS

### 3.1 Muscle atrophy due to hindlimb immobilization is correlated with mitochondrial dysfunction

**Figure 1:**
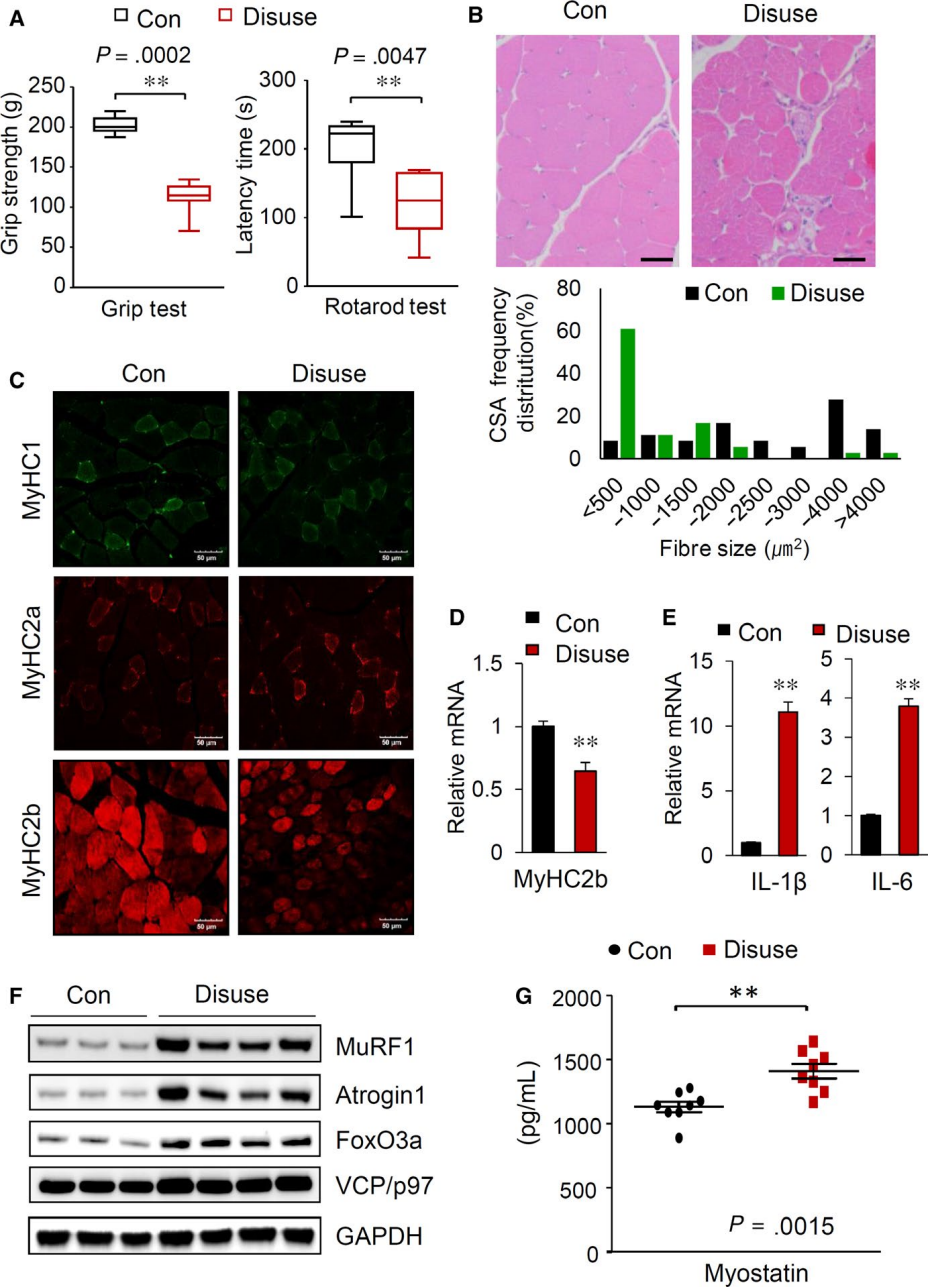
Muscle disuse generated by hindlimb immobilization in mice induces muscle atrophy. A, Grip strength measurements (left) and rotarod test results (right) of the control and hindlimb‐immobilized mice (n = 8). B, A reduction in muscle mass induced by hindlimb immobilization. H&E staining of GA muscle sections (top). Scale bars: 50 μm. Distribution of GA muscle fibres in cross‐sectional areas (bottom). C, Muscle sections prepared from GA muscles of the control and hindlimb‐immobilized mice were visualized with immunostaining against MyHC I, MyHC IIa and MyHC IIb. Scale bars: 50 μm. D, Relative mRNA levels of MyHC2B in the GA muscle (n = 8). E, Changes in the mRNA levels of IL‐1β and IL‐6 in the GA muscle (n = 8). F, Alterations in proteins related to muscle atrophy. Immunoblot analysis of the indicated proteins in the GA muscle. G, Serum myostatin levels in the control and hindlimb‐immobilized mice (n = 8). Serum myostatin levels (n = 8/group) were quantified by using a GDF8/myostatin Quantikine ELISA kit (R&D Systems) according to the manufacturer's instructions. All results are representative of more than three independent experiments. The data represent the mean ± SEM. *P* values < .05 obtained from Student's* t* tests were considered significant

To elucidate the underlying mechanisms of muscle atrophy, we first established a mouse model of skeletal muscle atrophy, which was generated by hindlimb immobilization (disuse) for 5 days. As expected, grip strength, endurance on the rotarod and muscle fibre diameter were decreased in the hindlimb‐immobilized mice compared with the control mice (Figure 1A,B). There are four distinct fibre types in mammalian skeletal muscles: oxidative slow‐twitch type 1 fibres, oxidative fast‐twitch type 2A fibres and glycolytic fast‐twitch type 2X fibres and 2B fibres, each containing different myosin heavy chain (MyHC) isoforms, referred to as MyHC1, MyHC2a, MyHC2x and MyHC2b, respectively.^15^ Campos's group and Bloemberg's group showed that type 2B fibres are predominant in the gastrocnemius (GA) muscle in mice.^16^, ^17^ Coincidently, when we observed muscle fibre distribution in the cross‐sectional area (CSA) of GA muscle by fluorescent staining, MyHC2b was the predominant isoform and was significantly reduced by hindlimb immobilization (Figure 1C). The MyHC1 and MyHC2a levels in GA muscle were low and did not change significantly following immobilization. This result was consistent with the significant reduction in the MyHC2b isoform expression in the GA muscles from the hindlimb‐immobilized mice (Figure 1D). A previously report showed that interleukin‐1 beta (IL‐1β) and interleukin‐6 (IL‐6) are involved in the muscle protein degradation.^18^ Consistent with this finding, muscle immobilization resulted in an increase in the expression of IL‐1β and IL‐6 in the GA muscle (Figure 1E). Additionally, the MuRF1, MAFbx/atrogin 1 and FoxO3a levels were markedly increased in the GA muscles of hindlimb‐immobilized mice, and p97/VCP, an ATPase complex known to facilitate myofibrillar protein degradation,^19^ was slightly elevated (Figure 1F). Moreover, we observed a significant increase in the serum myostatin levels in disuse mice compared with control mice (Figure 1G).

**Figure 2:**
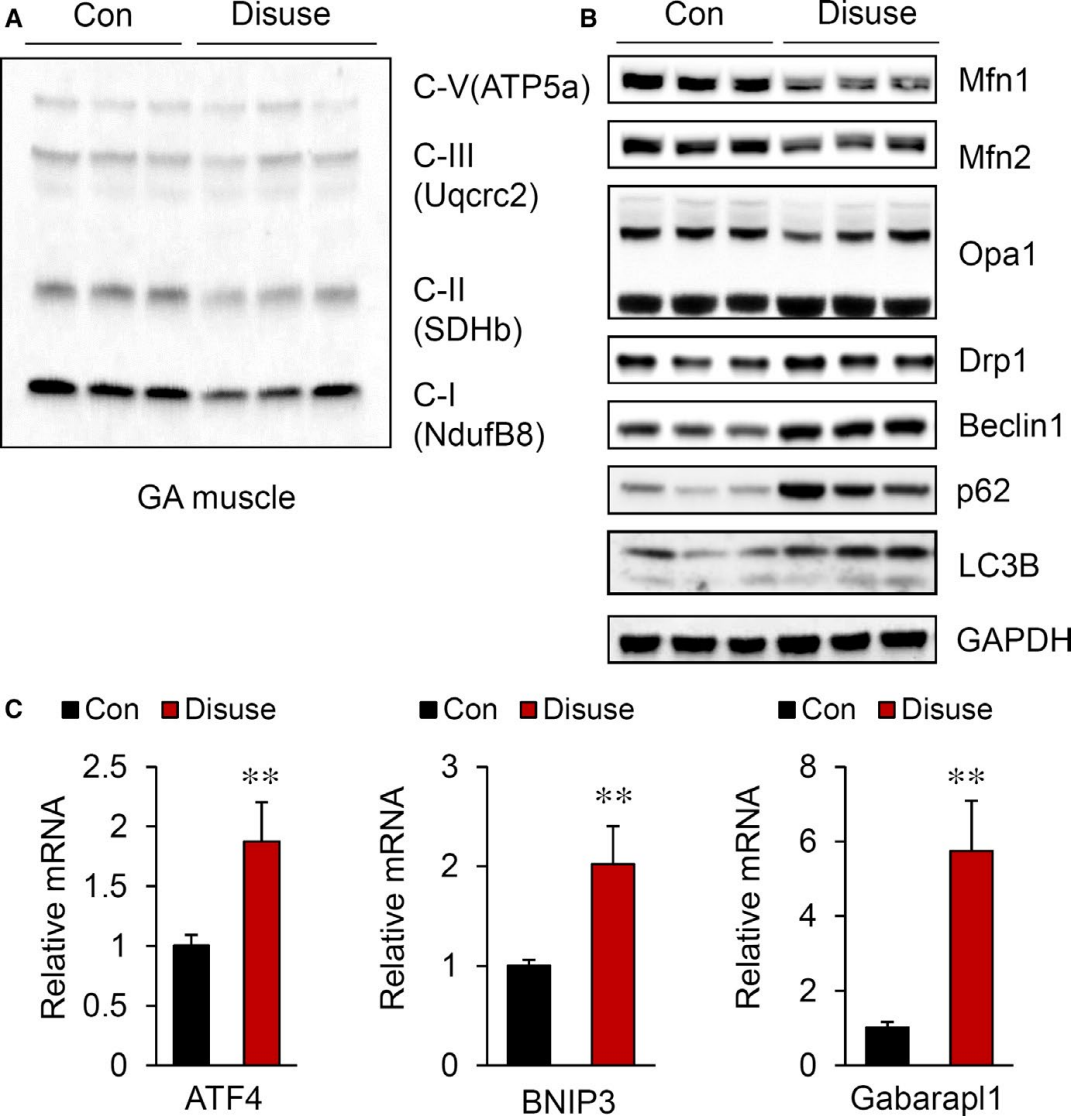
Disturbance in the balance of mitochondrial dynamics by hindlimb immobilization. Western blot analysis of mitochondrial OXPHOS complex subunits (A) and mitochondrial fusion and fission marker proteins (B) in the GA muscle. C, Relative mRNA levels of ATF4, BNIP3 and Gabarapl1 in the GA muscle (n = 8). All results are representative of more than three independent experiments. The data represent the mean ± SEM. *P* values < .05 obtained from Student's *t* tests were considered significant

To confirm whether muscle atrophy induced by immobilization is accompanied by mitochondrial dysfunction, we next performed Western blot analysis to measure the expression of different mitochondrial oxidative phosphorylation (OXPHOS) complex subunits. As shown in Figure 2A, the expression of OXPHOS components, especially those belonging to complex II, was markedly reduced in the GA muscles of hindlimb‐immobilized mice. Lecker S et al previously defined the genes that were down or up‐regulated in muscle atrophy as “atrogins”.^20^ Indeed, these electron transport chain complex proteins were shown to be atrogins that are negatively correlated with muscle atrophy.

Mitochondrial function is maintained by a mitochondrial quality control system. Therefore, we next measured whether proteins related to the mitochondrial quality control systems were changed. Mfn1, Mfn2 and the long isoform of OPA1 were significantly decreased (Figure 2B). Consistent with these findings, the levels of Drp1 and autophagy‐related genes, such as LC3B, SQSTM1/p62 and Beclin 1, were strongly up‐regulated in immobilized muscles. Additionally, activating transcription factor 4 (ATF4), a transcription factor that responds to mitochondrial stress 21; Bcl‐2/adenovirus E1B 19 kD‐interacting protein 3 (Bnip3); and GABA receptor‐associated protein‐like 1 (Gabarapl1), which are associated with mitophagy, were significantly induced in disuse mice (Figure 2C). Based on these results, we verified that the reduction in mitochondrial functions is closely related to muscle wasting.

### 3.2 CCCP‐induced mitochondrial dysfunction accompanies muscle atrophy in C2C12 myotubes

**Figure 3:**
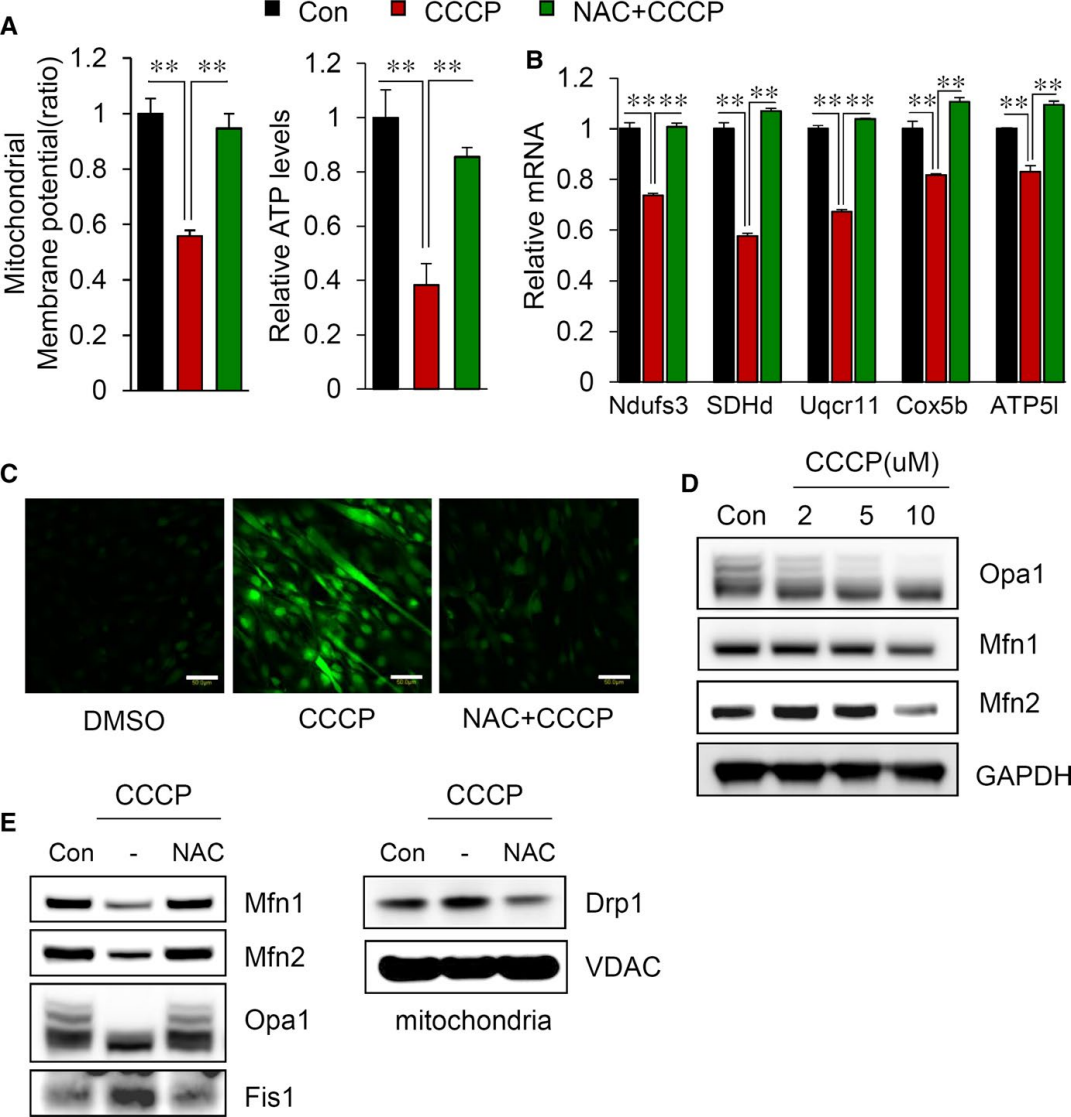
CCCP treatment causes an imbalance in mitochondrial dynamics in C2C12 myotubes. A, The mitochondrial membrane potential was evaluated as described in the Materials and Methods (left). Intracellular ATP levels were measured in C2C12 myotubes treated with 10 μmol/L CCCP in the presence or absence of 2 mmol/L NAC (right). ATP levels were estimated by using an ATP colorimetric/fluorometric assay kit (Abcam, ab83355) according to the manufacturer's instructions (B) Changes in the mRNA levels of mitochondrial OXPHOS complex subunits after CCCP treatment (C) Measurement of ROS in C2C12 myotubes as described in the Materials and Methods. Scale bars = 50 μm. D, Changes in the expression of proteins related to mitochondrial fusion and fission. E, Immunoblot analyses of proteins related to mitochondrial dynamics in C2C12 myotubes. All results are representative of more than three independent experiments. The data represent the mean ± SEM. *P* value < .05 obtained by one‐way ANOVA was considered statistically significant

To understand the molecular mechanisms by which mitochondrial dysfunction causes muscle atrophy, we used fully differentiated C2C12 myotubes that were treated with CCCP. As expected, the mitochondrial membrane potential and total ATP level were substantially reduced along with the reduction in the mitochondrial electron transport chain complex proteins in CCCP‐treated myotube cells (Figure 3 A,B). Mitochondrial dysfunction is known to increase intracellular ROS generation.^22^, ^23^ As shown in Figure 3C, the intracellular ROS levels were drastically elevated and the reduced by pretreatment with the antioxidant N‐acetylcysteine (NAC) in C2C12 myotubes. Next, we measured the proteins related to the mitochondrial dynamics and found that the levels of Mfn1, Mfn2 and the long form of OPA1 gradually decreased, while the expression of Fis1 increased upon treatment with CCCP and these effects were rescued by pretreatment with the antioxidant N‐acetylcysteine (NAC) in C2C12 myotubes (Figure 3D,E). Notably, the abnormal elevation of ROS shifted the mitochondrial dynamic balance towards mitochondrial fission.

**Figure 4:**
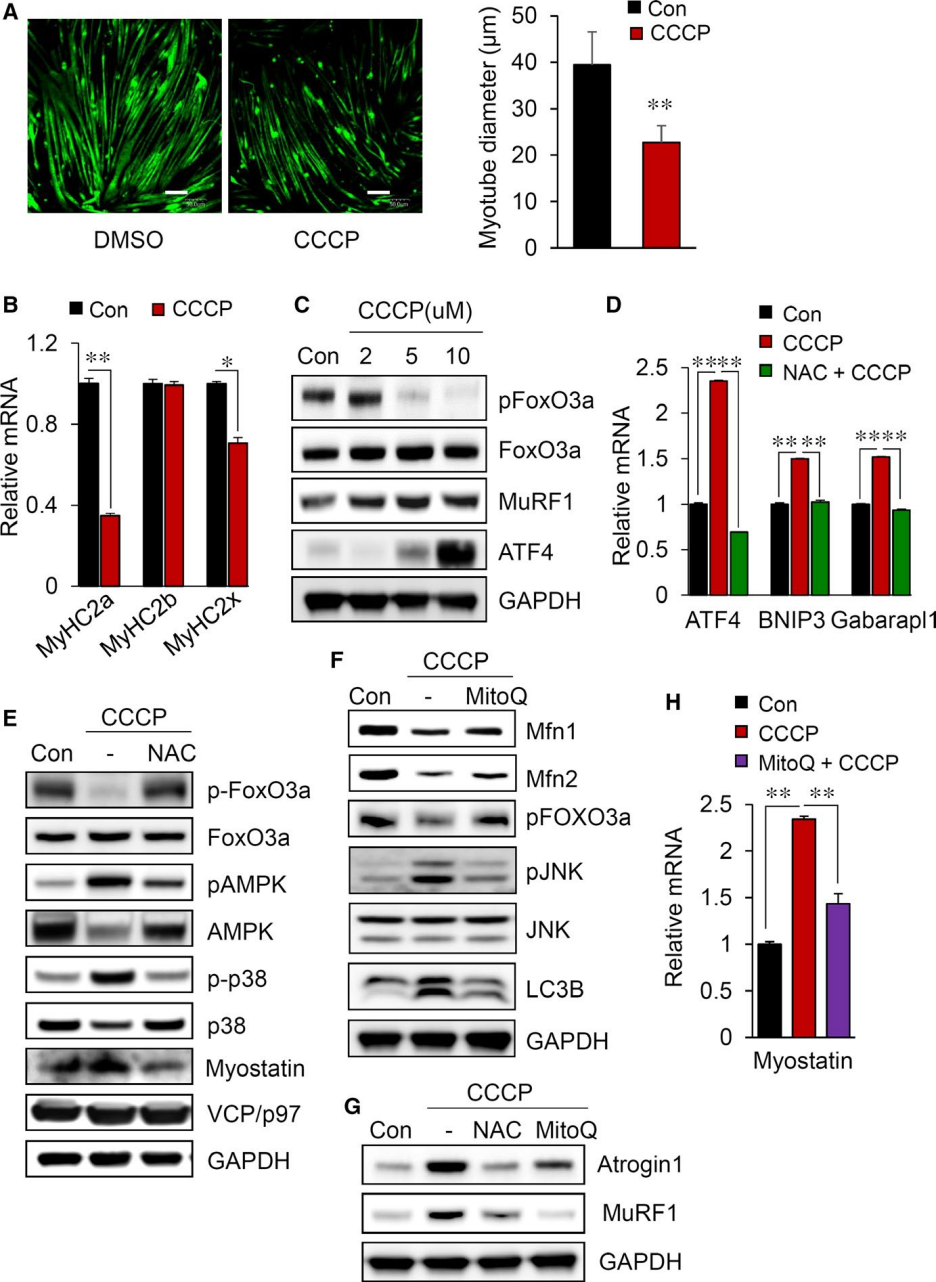
CCCP treatment induces muscle atrophy in C2C12 myotubes. A, Immunofluorescence staining for the whole myosin heavy chain in cells (left). Scale bars = 50 μm. Measurement of the diameter of myotubes in the CCCP‐treated cells compared with non‐treated cells (right). B, Reduction of the myosin heavy chain in C2C12 myotubes treated with 10 μmol/L CCCP in the presence or absence of 2 mmol/L NAC. Quantitative real‐time PCR analysis of MyHC isoforms (2A, 2B and 2X). C, Changes in the expression of muscle atrophy markers in myotubes. D, Relative mRNA levels of ATF4, BNIP3 and Gabarapl1 in CCCP‐treated C2C12 myotubes. E, Immunoblot analysis of proteins related to mitochondrial dynamics or muscle atrophy in C2C12 myotubes. (F,G) Immunoblot analysis of proteins related to mitochondrial dynamics or muscle atrophy in C2C12 myotubes treated with 10 μmol/L CCCP in the presence or absence of 0.5 μmol/L MitoQ. H, Relative mRNA levels of myostatin in C2C12 myotubes treated with 10 μmol/L CCCP in the presence or absence 0.5 μmol/L MitoQ. All results are representative of more than three independent experiments. The data represent the mean ± SEM. *P* values < .05 obtained by Student's *t* tests or one‐way ANOVA were considered statistically significant

We next examined whether the mitochondrial dysfunction as a result of CCCP induces muscle atrophy. As shown in Figure 4A and 4, the diameter of myotubes and the expression of MyHC were decreased by CCCP treatment. Coincidently, the levels of AMPK, MuRF1 and FoxO3a activity were significantly increased in the cells treated with CCCP (Figure 4C,E). In addition, we found that atrogins related to muscle atrophy, such as ATF4, myostatin and Gabarapl1 were significantly activated, and all these effects were attenuated by NAC (Figure 4D,E). To clarify the effects of mitochondrial dysfunction and the subsequent elevation of intracellular ROS on muscle atrophy, we specifically pretreated C2C12 myotubes with the mitochondria‐targeted antioxidant Mito Q. Pretreatment with Mito Q rescued mitochondrial fusion (reduction in mitophagy), and the subsequent reduction in muscle atrophy markers including FoxO3a, MuRF1, atrogin‐1 and myostatin (Figure 4F,G and H). As shown in Figure 4G, not only NAC but also the mitochondria‐specific antioxidant Mito Q could effectively ameliorate muscle atrophy induced by CCCP treatment. Therefore, the abnormal elevation of ROS as a result of mitochondrial dysfunction seems to be associated with muscle atrophy.

### 3.3 A reduction in mitochondrial Yme1L specifically induces muscle atrophy

**Figure 5:**
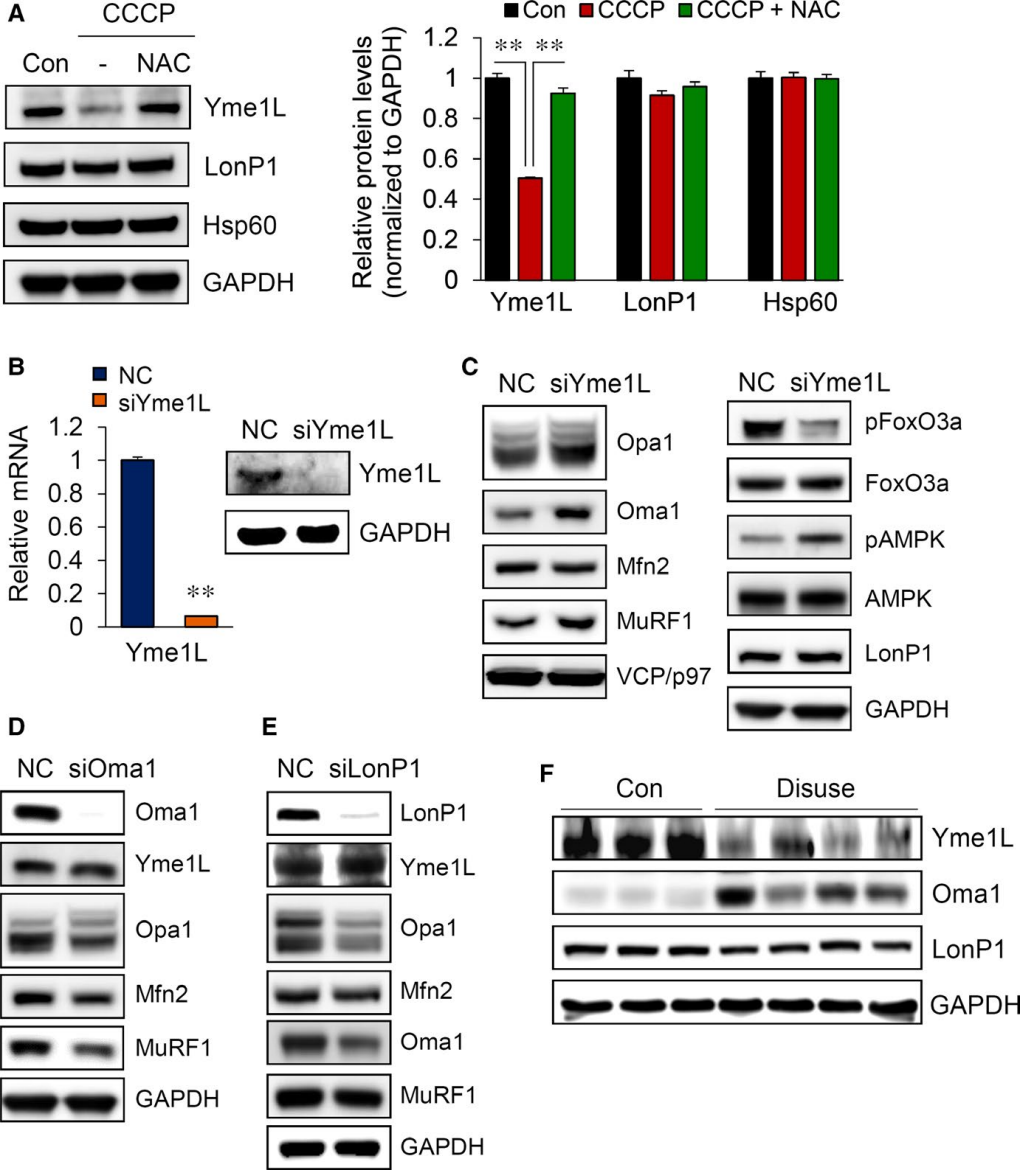
Knock‐down of Yme1L specifically causes muscle atrophy. A, Reduction of Yme1L following CCCP treatment. C2C12 myotubes were treated with 10 μmol/L CCCP in the presence or absence of 2 mmol/L NAC for 24 h. Western blot analyses are shown. B, Yme1L mRNA and protein levels were determined in 6‐day differentiated myotubes after transfection. C, The expression of proteins related to mitochondrial dynamics was analysed by Western blot analyses of 6‐day differentiated myotubes after transfection. (D and E) The expression of proteins related to mitochondrial fusion and muscle atrophy pathways was analysed by Western blots after siOma1 (D) or siLonP1 (E) transfection. F, Immunoblot analyses of Yme1L, Oma1 and LonP1 in the GA muscle. All results are representative of more than three independent experiments. The data represent the mean ± SEM. *P* values < .05 obtained from one‐way ANOVA were considered significant

We further examined the mechanisms by which elevated ROS causes muscle atrophy. The mitochondrial chaperones and proteases are essential to maintain proper mitochondria functions under stress conditions.^9^ Therefore, we examined the relationships between the proteases involved in mitochondrial quality control and muscle atrophy. As shown in Figure 5A, the expression levels of LonP1 and Hsp60 were almost unchanged in our experiment; however, the expression of Yme1L was significantly reduced in CCCP‐treated myotubes. In addition, these alterations were abolished by NAC pretreatment. To further directly analyse the effects of Yme1L depletion, we transfected *si*RNA specific for Yme1L into C2C12 myotube cells. We first showed that the expression of Yme1L was almost completely diminished (Figure 5B) and that the depletion of Yme1L increased the short form of Opa1, which was accompanied by an elevation in Oma1 expression, as previously described (Figure 5C).^12^ To determine whether the loss of Yme1L is related to muscle atrophy, we investigated alterations in proteins involved in muscle wasting. Intriguingly, we observed that Yme1L knock‐down substantially induced the activation of AMPK and FoxO3a and concomitantly increased MuRF1 expression. Oma1 is known to be responsible for the formation of s‐Opa1 when Yme1L is absent.^24^, ^25^ Accordingly, the loss of Oma1 significantly reduced not only the formation of the short form of Opa1 but also level of MuRF1; however, the levels of Yme1L itself were not altered in *si*Oma1‐transfected cells (Figure 5D). To test whether the loss of Yme1L specifically induces muscle atrophy, we transfected CCCP‐treated myotubes with *si*RNA targeting LonP1. In contrast to the loss of Yme1L, the depletion of LonP1 did not alter the expression of MuRF1 (Figure 5E). To confirm the relationship between the loss of Yme1L and muscle atrophy, we examined the expression level of Yme1L in the disused mice. As shown in Figure 5F, Yme1L was drastically reduced: consequently, Oma1 was significantly increased, whereas LonP1 was not altered in immobilized mice.

### 3.4 Yme1L loss of function results in the upregulation of myostatin in myotubes

**Figure 6:**
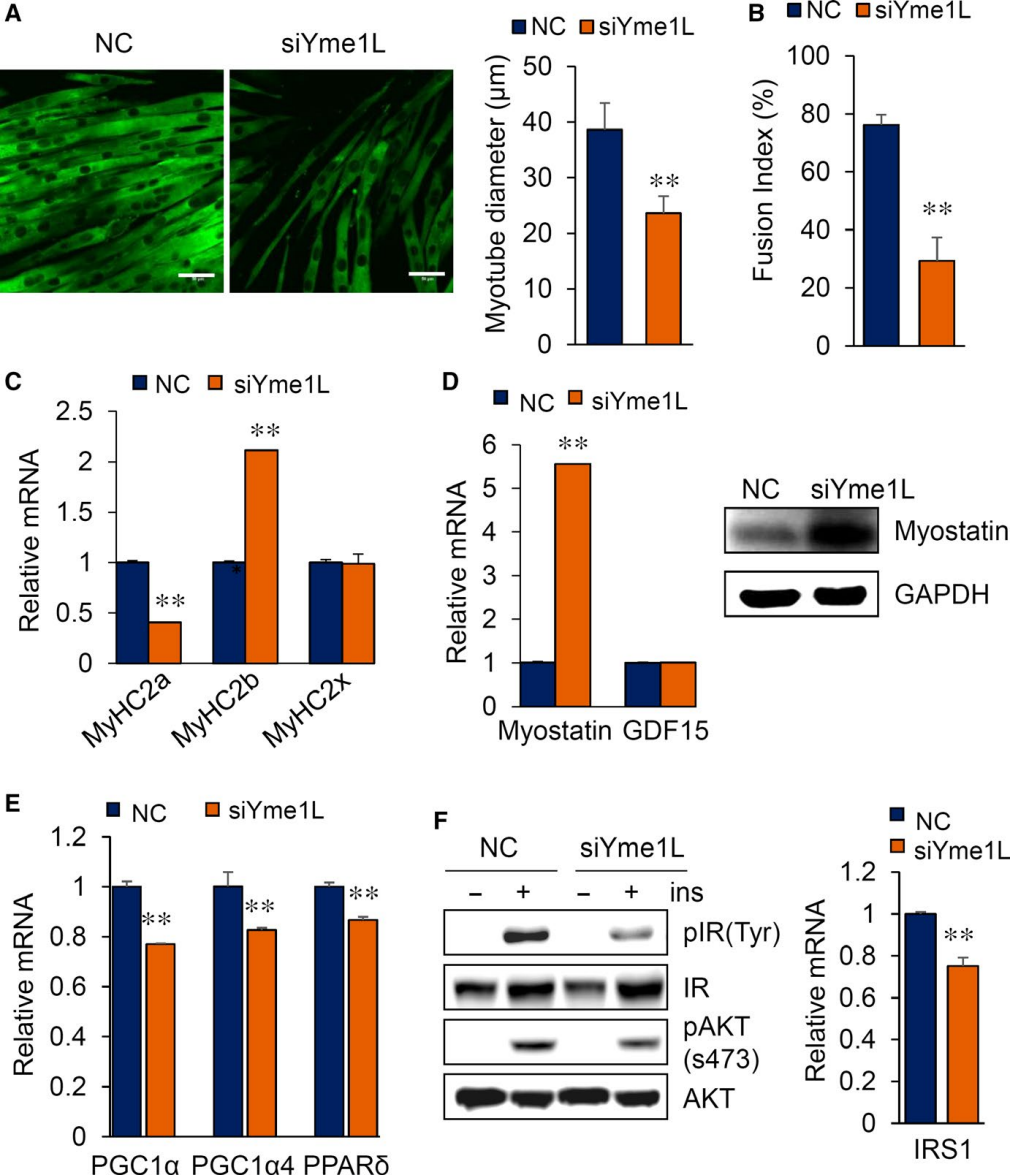
A reduction in Yme1L increases myostatin and perturbs insulin signalling. A, A reduction in MyHC following the depletion of Yme1L. Immunofluorescence staining for the myosin heavy chain in siRNA‐transfected cells (left). Scale bars = 50 μm. Analysis of the diameter of the myotubes (right). B, Calculation of the myotube fusion index as a percentage of the nuclei within a differentiated myotube containing more than three nuclei among the total nuclei (n = 4). C, Quantitative real‐time PCR analysis of MyHC isoforms (2A, 2B and 2X). D, Knock‐down of Yme1L elevated myostatin expression. Relative mRNA levels of myostatin and GDF15 in siRNA‐transfected C2C12 myotubes. E, Quantitative real‐time PCR analysis of PGC1α, PGC1α4 and PPARδ. F, Impairment of insulin activity by Yme1L depletion. Phosphorylated and total IR and Akt protein levels were determined by Western blotting (left). Relative mRNA levels of IRS1 (right). All results are representative of more than three independent experiments. The data represent the mean ± SEM. *P* values < .05 obtained from Student's *t* tests were considered significant

To further elucidate the role of Yme1L in muscle atrophy, we determined the morphological changes in myotubes after the transient knock‐down of Yme1L. As shown in Figure 6A and 6, the thickness of the myotubes and the myotube fusion index were significantly reduced by the depletion of Yme1L. We next analysed the expression patterns of MyHC isoforms in *si*Yme1L‐transfected cells. Consistent with the morphological changes, the expression of MyHC2a was significantly decreased, consistent with the observed results in the CCCP‐treated myotubes (Figure 6C). Interestingly, myostatin expression but not GDF15 expression was strongly increased by the transient knock‐down of Yme1L (Figure 6D). These findings were consistent with the down‐regulation of genes responsible for mitochondrial biogenesis and muscle protein synthesis, such as peroxisome proliferator‐activated receptor‐gamma coactivator 1 alpha (PGC1α), PGC1α4 and peroxisome proliferator‐activated receptors delta (PPARδ), observed in myotubes following Yme1L knock‐down (Figure 6E). Previously, McFarlane *et al* 26 reported that myostatin causes muscle atrophy via the repression of the IGF‐1/PI3K/Akt pathway. Thus, we next investigated whether the depletion of Yme1L affects Akt signalling. As shown in Figure 6F, Yme1L knock‐down inhibited Akt phosphorylation in differentiated myotubes. Intriguingly, the depletion of Yme1L also inhibited insulin‐induced insulin receptor signalling and reduced insulin receptor substrate‐1 (IRS‐1) expression (Figure 6F). These results suggest that the ablation of Yme1L alone causes muscle atrophy and disrupts insulin signalling, which may consequently lead to insulin resistance in atrophy‐induced muscle cells.

## 4 DISCUSSION

In recent decades, the importance of mitochondrial quality control in muscle atrophy has increased. Notably, the decline of mitochondrial function and the accompanying elevation of ROS have been gradually accepted as one of the hallmarks of ageing; however, the underlying mechanisms have not been fully elucidated. In the present study, we confirmed the causal relationship between mitochondrial dysfunction and skeletal muscle atrophy and showed that the loss of the mitochondrial i‐AAA protease Yme1L directly induces muscle atrophy in C2C12 myotubes via disruption of the balance in the mitochondrial quality control system. Here, we suggest that Yme1L is a key regulator in maintaining healthy muscle based on our results from siYme1L‐transfected myotubes; (a) altering mitochondrial dynamics towards mitochondrial fission via the elevation of Oma1 and the accumulation of the short form of Opa1; (b) reducing quantity of myotubes and the subsequent muscle hypotrophy; (c) inducing myostatin and the activation of the FoxO3a‐mediated ubiquitin‐proteasome system; and (d) down‐regulating the genes responsible for muscle protein synthesis and mitochondrial biogenesis, such as PGC‐1α4 and PGC‐1α. Indeed, the depletion of Yme1L significantly induced various effects that led to muscle wasting.

The cleavage of the long form of Opa1 to the short form is a critical regulatory step in balancing mitochondrial fusion and fission. Two mitochondrial proteases, namely, Yme1L and Oma1, cleave Opa1. While Yme1L constitutively cleaves Opa1 under normal conditions and is responsible for the maintenance of almost equimolar concentrations between L‐Opa1 and s‐Opa1, Oma1 is up‐regulated under stress conditions and converts Opa1 to the short form.^12^ We showed that the loss of Yme1L specifically results in muscle atrophy via the elevation of Oma1 and the concomitant accumulation of the short form of Opa1; however, the depletion of LonP1 in C2C12 myotubes did not directly induce muscle atrophy (Figure 5E). LonP1 is a major protein in the mtUPR and is important for mitochondrial biogenesis.^27^ Previously, Wagatsuma A *et al* 28 reported that LonP1 is reduced in hindlimb unloading mice; nevertheless, in our work the loss of LonP1 did not trigger muscle atrophy. LonP1 is likely reduced when mitochondria are massively decreased by prolonged muscle unloading; however the reduction in LonP1 itself does not accompany muscle atrophy. In contrast, reduction in Yme1L seems directly to induce protein breakdown in muscle cells via an imbalance in mitochondrial dynamics. Indeed, Yme1L participates in various processes to maintain healthy mitochondria, including the regulation of mitophagy by controlling Oma1 and s‐Opa1.^29^ Yme1L is involved in the assembly of mitochondrial respiratory chain subunits, including F1F0‐ATPase, Cox4 and ND1,^30^ and it removes damaged or misfolded mitochondrial inner membrane proteins.^31^ Recently, Yme1L was shown to participate in the degradation of Tom22, a receptor involved in mitochondrial protein import that is located in the mitochondrial outer membrane.^32^ Despite the important role of Yme1L in the maintenance of mitochondrial functions, the regulation of Yme1L expression is still unclear. Previously, Hori O *et al* reported that the accumulation of unassembled proteins in the mitochondrial inner membrane triggers Yme1L expression 33; however, few reports have addressed the regulation of Yme1L itself. Figure 5A shows that the reduction in Yme1L is closely related to the abnormal elevation of ROS. Here, we suggest that conditions which induce ROS accumulation result in the decrease of the expression of Yme1L, thereby initiating muscle atrophy. When muscle atrophy is initiated, dysfunctional mitochondria accumulate and, in turn, result in an additional increase in ROS, forming a vicious cycle to worsen muscle atrophy.

Myostatin is a negative regulator of muscle mass, and its overexpression reduces muscle mass in adult mice and rats^34^ as well as in humans.^35^ However, the regulation of myostatin expression has not been clearly elucidated. Notably, our data showed that the loss of Yme1L drastically increases myostatin in C2C12 myotubes (Figure 6D). The activation of myostatin following Yme1L depletion seems to be the important mechanisms by which an imbalance in mitochondrial dynamics induces muscle atrophy, but more studies are required to understand the precise mechanisms by which mitochondrial dynamics regulates myostatin expression.

In summary, our results demonstrated the correlations between balanced mitochondrial dynamics and the maintenance of mass and skeletal muscle function, and we suggest that Yme1L may play a pivotal role in the regulation of muscle mass by controlling mitochondrial dynamics. The loss of Yme1L activates AMPK but represses IGF‐1/Akt signalling, in turn activating FoxO3a. In addition, elevation of myostatin together facilitates muscle fibre breakdown which concomitantly leads to muscle atrophy. Further studies are required to elucidate whether Yme1L is decreased in sarcopenic atrophy in mice and humans.

## CONFLICT OF INTEREST

The authors declare that they have no conflict of interest.

## AUTHOR CONTRIBUTIONS

JHL designed this study and wrote the manuscript. YJL wrote the draft of this manuscript and mainly performed experiments and analysed data. GHK performed several experiments and analysed data. SIP discussed the results and commented on the manuscript.

## Data Availability

The data used to support the findings of this study are available from the corresponding author upon request.

## References

[1] Anton SD , Karabetian C , Naugle K , et al. Obesity and diabetes as accelerators of functional decline: can lifestyle interventions maintain functional status in high risk older adults? Exp Gerontol. 2013;48:888‐897. 23832077 10.1016/j.exger.2013.06.007 PMC3817488

[2] Rom O , Reznick AZ . The role of E3 ubiquitin‐ligases MuRF‐1 and MAFbx in loss of skeletal muscle mass. Free Radic Biol Med. 2016;98:218‐230. 26738803 10.1016/j.freeradbiomed.2015.12.031

[3] Ost M , Coleman V , Kasch J , et al. Regulation of myokine expression: role of exercise and cellular stress. Free Radic Biol Med. 2016;98:78‐89. 26898145 10.1016/j.freeradbiomed.2016.02.018

[4] Rodriguez J , Vernus B , Chelh I , et al. Myostatin and the skeletal muscle atrophy and hypertrophy signaling pathways. Cell Mol Life Sci. 2014;71:4361‐4371. 25080109 10.1007/s00018-014-1689-x PMC11113773

[5] Bowen TS , Schuler G , Adams V . Skeletal muscle wasting in cachexia and sarcopenia: molecular pathophysiology and impact of exercise training. J Cachexia Sarcopenia Muscle. 2015;6:197‐207. 26401465 10.1002/jcsm.12043 PMC4575550

[6] Picca A , Calvani R , Bossola M , et al. Update on mitochondria and muscle aging: all wrong roads lead to sarcopenia. Biol Chem. 2018;399:421‐436. 29384724 10.1515/hsz-2017-0331

[7] Romanello V , Sandri M . Mitochondrial quality control and muscle mass maintenance. Front Physiol. 2015;6:422. 26793123 10.3389/fphys.2015.00422 PMC4709858

[8] Sebastian D , Palacin M , Zorzano A . Mitochondrial dynamics: coupling mitochondrial fitness with healthy aging. Trends Mol Med. 2017;23:201‐215. 28188102 10.1016/j.molmed.2017.01.003

[9] Voos W . Chaperone‐protease networks in mitochondrial protein homeostasis. Biochim Biophys Acta. 2013;1833:388‐399. 22705353 10.1016/j.bbamcr.2012.06.005

[10] Quiros PM , Langer T , Lopez‐Otin C . New roles for mitochondrial proteases in health, ageing and disease. Nat Rev Mol Cell Biol. 2015;16:345‐359. 25970558 10.1038/nrm3984

[11] Opalinska M , Jańska H . AAA proteases: guardians of mitochondrial function and homeostasis. Cells. 2018;7(10):163. 10.3390/cells7100163 PMC6210556 30314276

[12] Anand R , Wai T , Baker MJ , et al. The i‐AAA protease YME1L and OMA1 cleave OPA1 to balance mitochondrial fusion and fission. J Cell Biol. 2014;204:919‐929. 24616225 10.1083/jcb.201308006 PMC3998800

[13] Caron AZ , Drouin G , Desrosiers J , et al. A novel hindlimb immobilization procedure for studying skeletal muscle atrophy and recovery in mouse. J Appl Physiol. 1985;2009(106):2049‐2059. 10.1152/japplphysiol.91505.2008 19342435

[14] Hutter‐Saunders JA , Gendelman HE , Mosley RL . Murine motor and behavior functional evaluations for acute 1‐methyl‐4‐phenyl‐1,2,3,6‐tetrahydropyridine (MPTP) intoxication. J Neuroimmune Pharmacol. 2012;7:279‐288. 21431472 10.1007/s11481-011-9269-4 PMC3392900

[15] Schiaffino S , Reggiani C . Fiber types in mammalian skeletal muscles. Physiol Rev. 2011;91:1447‐1531. 22013216 10.1152/physrev.00031.2010

[16] Augusto V , Padovani C , Campos G . Skeletal muscle fiber types in C57BL6J mice. Braz J morphol Sci. 2004;21:89‐94.

[17] Bloemberg D , Quadrilatero J . Rapid determination of myosin heavy chain expression in rat, mouse, and human skeletal muscle using multicolor immunofluorescence analysis. PLoS ONE. 2012;7:e35273. 22530000 10.1371/journal.pone.0035273 PMC3329435

[18] Tsujinaka T , Fujita J , Ebisui C , et al. Interleukin 6 receptor antibody inhibits muscle atrophy and modulates proteolytic systems in interleukin 6 transgenic mice. J Clin Invest. 1996;97:244‐249. 8550842 10.1172/JCI118398 PMC507086

[19] Piccirillo R , Goldberg AL . The p97/VCP ATPase is critical in muscle atrophy and the accelerated degradation of muscle proteins. EMBO J. 2012;31:3334‐3350. 22773186 10.1038/emboj.2012.178 PMC3411080

[20] Lecker SH , Jagoe RT , Gilbert A , et al. Multiple types of skeletal muscle atrophy involve a common program of changes in gene expression. FASEB J. 2004;18:39‐51. 14718385 10.1096/fj.03-0610com

[21] Quiros PM , Prado MA , Zamboni N , et al. Multi‐omics analysis identifies ATF4 as a key regulator of the mitochondrial stress response in mammals. J Cell Biol. 2017;216:2027‐2045. 28566324 10.1083/jcb.201702058 PMC5496626

[22] Sadeghi A , Seyyed Ebrahimi SS , Golestani A , et al. Resveratrol ameliorates palmitate‐induced inflammation in skeletal muscle cells by attenuating oxidative stress and JNK/NF‐kappaB pathway in a SIRT1‐independent mechanism. J Cell Biochem. 2017;118:2654‐2663. 28059488 10.1002/jcb.25868

[23] Chen T , Zhu J , Wang YH , et al. ROS‐mediated mitochondrial dysfunction and er stress contribute to compression‐induced neuronal injury. Neuroscience. 2019;416:268‐280. 31425734 10.1016/j.neuroscience.2019.08.007

[24] Baker MJ , Lampe PA , Stojanovski D , et al. Stress‐induced OMA1 activation and autocatalytic turnover regulate OPA1‐dependent mitochondrial dynamics. EMBO J. 2014;33:578‐593. 24550258 10.1002/embj.201386474 PMC3989652

[25] Rainbolt TK , Lebeau J , Puchades C , et al. Reciprocal degradation of YME1L and OMA1 adapts mitochondrial proteolytic activity during stress. Cell Rep. 2016;14:2041‐2049. 26923599 10.1016/j.celrep.2016.02.011 PMC4785047

[26] McFarlane C , Plummer E , Thomas M , et al. Myostatin induces cachexia by activating the ubiquitin proteolytic system through an NF‐kappaB‐independent, FOXO1‐dependent mechanism. J Cell Physiol. 2006;209:501‐514. 16883577 10.1002/jcp.20757

[27] Bota DA , Davies KJ . Mitochondrial Lon protease in human disease and aging: Including an etiologic classification of Lon‐related diseases and disorders. Free Radic Biol Med. 2016;100:188‐198. 27387767 10.1016/j.freeradbiomed.2016.06.031 PMC5183306

[28] Wagatsuma A , Kotake N , Kawachi T , et al. Mitochondrial adaptations in skeletal muscle to hindlimb unloading. Mol Cell Biochem. 2011;350:1‐11. 21165677 10.1007/s11010-010-0677-1

[29] Cesnekova J , Rodinova M , Hansikova H , et al. Loss of mitochondrial AAA proteases AFG3L2 and YME1L impairs mitochondrial structure and respiratory chain biogenesis. Int J Mol Sci. 2018;19(12):3930. 10.3390/ijms19123930 PMC6321463 30544562

[30] Stiburek L , Cesnekova J , Kostkova O , et al. YME1L controls the accumulation of respiratory chain subunits and is required for apoptotic resistance, cristae morphogenesis, and cell proliferation. Mol Biol Cell. 2012;23:1010‐1023. 22262461 10.1091/mbc.E11-08-0674 PMC3302729

[31] Shi H , Rampello AJ , Glynn SE . Engineered AAA+ proteases reveal principles of proteolysis at the mitochondrial inner membrane. Nat Commun. 2016;7:13301. 27786171 10.1038/ncomms13301 PMC5095350

[32] Wu X , Li L , Jiang H . Mitochondrial inner‐membrane protease Yme1 degrades outer‐membrane proteins Tom22 and Om45. J Cell Biol. 2018;217:139‐149. 29138251 10.1083/jcb.201702125 PMC5748973

[33] Hori O , Ichinoda F , Tamatani T , et al. Transmission of cell stress from endoplasmic reticulum to mitochondria: enhanced expression of Lon protease. J Cell Biol. 2002;157:1151‐1160. 12082077 10.1083/jcb.200108103 PMC2173558

[34] Durieux AC , Amirouche A , Banzet S , et al. Ectopic expression of myostatin induces atrophy of adult skeletal muscle by decreasing muscle gene expression. Endocrinology. 2007;148:3140‐3147. 17395701 10.1210/en.2006-1500

[35] Lokireddy S , Mouly V , Butler‐Browne G , et al. Myostatin promotes the wasting of human myoblast cultures through promoting ubiquitin‐proteasome pathway‐mediated loss of sarcomeric proteins. Am J Physiol Cell Physiol. 2011;301:C1316‐ C1324. 21900687 10.1152/ajpcell.00114.2011

