# Supplementary material for: Down‐regulation of the mitochondrial i‐AAA protease Yme1L induces muscle atrophy via FoxO3a and myostatin activation
Source: J Cell Mol Med. 2019 Nov 14;24(1):899–909. doi: 10.1111/jcmm.14799 (PMC6933342; doi:10.1111/jcmm.14799)
Supplement: Supplementary file 1 [file JCMM-24-899-s001.docx]

**Supporting Information**

**Table S1. Primer list used for qPCR.**

| Primer name | Direction | Sequence |
| --- | --- | --- |
| MYH2A | Forward | AATCGAGGCCCAGAATAGGC |
|  | Reverse | AATCGAGGCCCAGAATAGGC |
| MYH2B | Forward | TGGCCGAGCAAGAGCTAC |
|  | Reverse | TTGATGAGGCTGGTGTTCTGG |
| MYH2X (MYH1) | Forward | AATCAAAGGTCAAGGCCTACAA |
|  | Reverse | GAATTTGGCCAGGTTGACAT |
| MYH7 | Forward | ACTGTCAACACTAAGAGGGTCA |
|  | Reverse | TTGGATGATTTGATCTTCCAGGG |
| IL-1b | Forward | CGCAGCAGCACATCAACAAG |
|  | Reverse | GTGCTCATGTCCTCATCCTG |
| IL-6 | Forward | TAGTCCTTCCTACCCCAATTTCC |
|  | Reverse | TTGGTCCTTAGCCACTCCTTC |
| Yme1l1 | Forward | ATTCTGCGGTAGACCCTGTC |
|  | Reverse | CAACCACTTCCTGTAACTCTTG |
| LonP1 | Forward | ATGACCGTCCCGGATGTGT |
|  | Reverse | CCTCCACGATCTTGATAAAGCG |
| CLPP | Forward | CAGTCTGAAAGCAACAAGAAGC |
|  | Reverse | CTGCATTGTGTCGTAGATGG |
| Hsp60 | Forward | ACGATCTATTGCCAAGGAGG |
|  | Reverse | TCAGGGGTTGTCACAGGTTT |
| Hsp70 | Forward | ATGGCTGGAATGGCCTTAGC |
|  | Reverse | CCCAAATCAATACCAACCACTG |
| Myostatin(Mstn) | Forward | TAACCTTCCCAGGACCAGGA |
|  | Reverse | CACTCTCCTGAGCAGTAATT |
| GDF15 | Forward | GAGCTACGGGGTCGCTTC |
|  | Reverse | GGGACCCCAATCTCACCT |
| PGC1α | Forward | AGCCGTGACCACTGACAACGAG |
|  | Reverse | GCTGCATGGTTCTGAGTGCTAAG |
| PGC1α4 | Forward | TCACACCAAACCCACAGAAA |
|  | Reverse | CTGGAAGATATGGCACAT |
| PPARδ | Forward | ACGCACCCTTTGTCATCCA |
|  | Reverse | TTCCACACCAGGCCCTTCT |
| GAPDH | Forward | CATGGCCTTCCGTGTTCCTA |
|  | Reverse | CCTGCTTCACCACCTTCTTGAT |
